# Global gene expression profiling of cells overexpressing SMC3

**DOI:** 10.1186/1476-4598-4-34

**Published:** 2005-09-12

**Authors:** Giancarlo Ghiselli, Chang-Gong Liu

**Affiliations:** 1 Department of Pathology and Cell Biology, Thomas Jefferson University, 1020 Locust Street, Philadelphia, PA 19107, USA; 2 Kimmel Cancer Center, Thomas Jefferson University, 1020 Locust Street, Philadelphia, PA 19107, USA; 3 Department of Microbiology and Immunology, Thomas Jefferson University, 1020 Locust Street, Philadelphia, PA 19107, USA

## Abstract

**Background:**

The Structural Maintenance of Chromosome 3 protein (SMC3) plays an essential role during the sister chromatid separation, is involved in DNA repair and recombination and participates in microtubule-mediated intracellular transport. SMC3 is frequently elevated in human colon carcinoma and overexpression of the protein transforms murine NIH3T3 fibroblasts. In order to gain insight into the mechanism of SMC3-mediated tumorigenesis a gene expression profiling was performed on human 293 cells line stably overexpressing SMC3.

**Results:**

Biotinylated complementary RNA (cRNA) was used for hybridization of a cDNAmicroarray chip harboring 18,861 65-mer oligos derived from the published dEST sequences. After filtering, the hybridization data were normalized and statistically analyzed. Sixty-five genes for which a putative function could be assigned displayed at least two-fold change in their expression level. Eighteen of the affected genes is either a transcriptional factor or is involved in DNA and chromatin related mechanisms whereas most of those involved in signal transduction are members or modulators of the ras-rho/GTPase and cAMP signaling pathways. In particular the expression of RhoB and CRE-BPa, two mediators of cellular transformation, was significantly enhanced. This association was confirmed by analyzing the RhoB and CRE-BPa transcript levels in cells transiently transfected with an SMC3 expression vector. Consistent with the idea that the activation of ras-rho/GTPase and cAMP pathways is relevant in the context of the cellular changes following SMC3 overexpression, gene transactivation through the related serum (SRE) and cAMP (CRE) *cis*-acting response elements was significantly increased.

**Conclusion:**

We have documented a selective effect of the ectopic expression of SMC3 on a set of genes and transcriptional signaling pathways that are relevant for tumorigenesis. The results lead to postulate that RhoB and CRE-BPa two known oncogenic mediators whose expression is significantly increased following SMC3 overexpression play a significant role in mediating SMC3 tumorigenesis.

## Introduction

The Structural Maintenance of Chromosome 3 protein (SMC3) is a key component of the nuclear multimeric protein complex named cohesin. This complex, which also includes SMC1, scc1 and scc3, forms joints between the replicating DNA strands and holds together the sister chromatids throughout G_2 _phase while opposing the splitting force exerted by the spindle microtubules [1]. In addition to its essential role in mitotic and meiotic chromosome segregation, SMC3 plays an important role in DNA recombination [2], is a component of the DNA damage repair mechanism [3] and is involved in the microtubule-based intracellular transport [4]. SMC3 expression is elevated in a large fraction of human colon carcinoma and in the intestinal tumors of mice genetically prone to develop polyps [5]. SMC3 expression level is controlled in intestinal epithelial cells through the APC/β-catenin/TCF4 transactivation pathway a signaling system that is almost invariably altered in colon carcinomas [6]. Furthermore NIH3T3 fibroblasts overexpressing SMC3 lose cell-cell contact inhibition, display anchorage-independent growth and form foci of transformation [5]. These findings support the idea that up-regulation of SMC3 expression is either permissive or sufficient to trigger cell transformation. The mechanism of SMC3-mediated cell transformation has however remained speculative.

In order to identify genes whose expression is affected by SMC3 overexpression, high-density oligonucleotide microarray chip harboring 18,861 human gene-specific oligonucleotides were hybridized with cRNA derived from 293 cells with different expression level of SMC3. The 293 cells are human embryonic kidney cells that have become immortalized following transformation by adenovirus type 5 [7] and display latent tumorigenicity [8]. This represent a well characterized model for human tumorigenesis that has been frequently utilized for *in vitro *and *in vivo *assessment of the oncogenic or tumor suppressor potential of a number of genes [9-13]. Statistical analysis of the microarray data has revealed that many of the genes affected by SMC3 overexpression in 293 cells are members or modulators of the ras-rho/GTPase family of proteins and of the cAMP signaling pathway. The analysis of the activity of a panel of reporter vectors monitoring different transactivation pathways further corroborates the idea that ras-rho/GTPase and cAMP response element binding proteins play a predominant role in orchestrating the cell changes subsequent to SMC3 overexpression. In particular RhoB and CRE-BPa, two major modulators of cellular transformation and response to genotoxic stress and whose level is significantly increased following SMC3 overexpression, may act as important mediators of SMC3 activity at cellular level.

## Results and Discussion

### A microarray analysis of the genome-wide effect of SMC3 overexpression identifies candidate genes mediating SMC3 tumorigenicity

**Figure 1:**
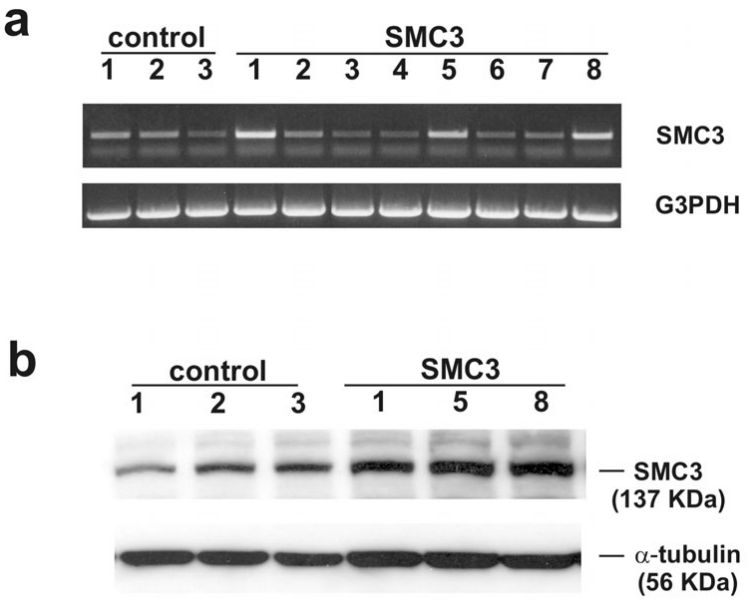
**Identification and characterization of 293 cells stably overexpressing human SMC3**. a): 293 cells were transfected with SMC3-pcDNA3.1 expression vector or the empty vector alone. Cell clones with stably integrated vector were selected in medium containing 500 μg/ml of G418 for three weeks. The expression of SMC3 and of the housekeeping gene G3DPH in drug-resistant clones was evaluated by semi-quantitative RT-PCR. b): SMC3 expression analysis was repeated on cells at later passages to confirm that clones stably overexpressing SMC3 were used. This time SMC3 expression was examined by Western immunoblotting. For this purpose cells were solubilized in lysis buffer and analyzed by 8% SDS-PAGE. After transfer of the proteins to nitrocellulose, SMC3 was immunodetected using a goat polyclonal antibody followed by ECL detection of the immunocomplex. The expression of α-tubulin was used to assess the inter-sample variability using nitrocellulose filters stripped of the SMC3 immunocomplexes.

The identification of genes that are affected by SMC3 up-regulation may provide important clues regarding the biology of this cohesin protein and shed light on the mechanism at the basis of the SMC3-induced tumorigenesis. Toward this end the changes in gene expression caused by sustained SMC3 overexpression were analyzed in fetal kidney 293 cells using a large microarray of human gene-specific oligonucleotides. Stable overexpression of SMC3 in these cells was assessed by semi-quantitative RT-PCR and the result confirmed in cells at a later division stage by Western immunoblotting (fig. 1). On the average we detected ~3-fold elevation of the SMC3 mRNA and protein levels. These values compare well with the 2-folds elevation of SMC3 transcript level measured in the microarray analysis (Table I). Of relevance is the fact that these changes in expression level are similar to those previously detected in a series of human colon carcinomas and in the intestinal polyps of APC^+/min ^mice or in NIH3T3 cells genetically engineered to ectopically express SMC3 [5]. A comparable increase in SMC3 expression has also been detected in liver metastatic cancer cells [14], in marrow stem cells exiting quiescence [15], in vascular endothelial cells following angiogenic stimulus [16], and in human T-cells infected with Varicella-Zoster [17] suggesting that maximal achievable SMC3 expression level is similar in different cell and tissues contexts. Interestingly these changes in transcriptional activity are not accompanied by significant changes in the expression of the other components of the cohesin complex consistent with the idea that SMC3 level is regulated independently from that of the other partner proteins.

**Table 1:** Significantly regulated genes in SMC3 overexpressing 293 cells. Human expressed sequence tags and genes with no known function are not included. Complete results of the array have been submitted to the EBI microarray database.

***Genes***	***SAM Score***	***Fold changes***
***Bamacan/SMC3***	***2.06***	***2.04***
		
*Extracellular Matrix*		
		
GPC3, glypican 3	2.55	2.49
lectin, mannose-binding, 1 like	2.05	2.13
HAS2, hyaluronan synthase 2	1.89	2.03
LAMB3, lamin, beta 3	-1.99	-3.76
		
*Transporter and ion channels*		
		
ATP6V1B1, ATPase, H^+ ^transporting V1 subunit B, kidney isoform	3.15	2.40
KCTD12, potassium channel tetramerisation domain 12	1.85	2.02
SLC7A10, solute carrier family 7 member 10, neutral amino acid transporter	-2.00	-2.00
		
*Metabolism*		
		
MIG12, MID1 interacting G12-like protein	2.58	2.65
CKB, creatine kinase, B chain	2.00	2.28
ECHDC3, enoyl Coenzyme A hydratase domain containing 3	2.25	2.20
ALDH1A3, aldehyde dehydrogenase 1 family, member A3	1.94	2.16
MOXD1, monooxygenase, DBH-like 1	2.53	2.05
TXNRD3, thioredoxin reductase 3	1.81	2.00
		
*Growth factors and receptors*		
		
LTBP2, latent transforming growth factor beta binding protein 2	1.95	2.68
GABRE, gamma-aminobutyric acid A receptor, epsilon	2.04	2.54
DLL1, delta-like 1, notch ligand	1.83	2.23
GDF9, growth differentiation factor 9	1.88	2.19
IGFBP7, insulin-like growth factor binding protein 7	1.81	2.15
GNRH1, gonadotropin-releasing hormone 1	2.23	2.04
IRAK1, interleukin-1 receptor associated kinase 1	-2.17	-2.13
EGFL3, EGF-like-domain, multiple 3	-2.20	-2.16
IL22RA1, interleukin 22 receptor, alpha 1	-2.22	-2.48
OR4D1, olfactory receptor, family 4, subfamily D, member 1	-1.91	-3.03
GRB7, growth factor receptor-bound protein 7	-2.20	-4.18
		
*Signal Transduction*		
		
ARHGEF4, Rho guanine nucleotide exchange factor 4	2.36	2.85
RGS14, regulator of G-protein signaling 14	2.17	2.57
RPS6KA5, ribosomal protein S6 kinase, polypeptide 5	2.09	2.46
PAK6, p21-activated kinase 6	2.16	2.38
RIN3, Ras and Rab interactor 3	1.86	2.19
RHOB, ras homolog gene family, member B	1.81	2.18
SCFD1, sec1 family domain containing 1	2.31	2.08
LRRK1, leucine-rich repeat kinase 1	2.16	2.06
RAB40B, GTP-binding protein 40B	1.91	2.15
PDE6B, phosphodiesterase 6B, cGMP-specific	2.02	2.09
MCF2L, MCF.2 cell line derived transforming sequence	-1.92	-2.16
S100A8, S 100 calcium binding protein A8, calgranulin A	-1.92	-2.86
ADCY2, adenylate cyclase 2	-1.92	-3.03
		
*Transcriptional factors*		
		
MEOX2, mesenchyme homeobox 2, growth arrest specific homeobox	2.14	3.96
IRF4, interferon regulatory factor 4	2.20	3.28
KHDRBS3, KH domain, RNA binding, signal transduction associated 3	2.24	2.62
PPARA, peroxisome proliferative activated receptor, alpha	2.22	2.50
CRE-BPa, cAMP responsive element binding protein, ATF2-like	1.94	2.37
NEK9, never-in-mitosis-gene a-related kinase 9	1.83	2.33
CREM, cAMP responsive element modulator	-1.87	-2.11
NCOA6, nuclear receptor coactivator 6	-1.88	-2.32
TXB21, T-box 21	-1.88	-2.42
POU3F1, POU domain, class 3, octamer-binding TF6	-2.21	-2.44
NKX6-1, NK6 transcriptional factor related, locus 1, homeobox 6A	-1.90	-2.53
CTNNA1, catenin alpha 1	-1.90	-2.57
BCL6B, B-cell CLL/lymphoma 6, member B zinc finger protein	-2.72	-2.79
ZNF236, zinc finger protein 236	-1.90	-3.11
		
*DNA repair, gene transcription*		
		
MHL3, mutL homolog 3	2.76	2.81
DNMT2, DNA cytosine-5-methyltransferase 2	1.88	2.07
ADPRTL2, ADP-ribosyltransferase 2	2.11	2.00
SNRPN, small nuclear ribonuclear polypeptide N	-2.25	-2.64
		
*Various*		
		
MYOM2, myomesin 2	2.77	5.10
NOPE, neighbor of Punc E11	2.53	2.56
C5, complement component 5	2.09	2.46
COCH, coagulation factor C homolog, cochlin	2.15	2.09
VMD2L1, vitelliform macular dystrophy 2-like 1	-2.17	-2.09
GRN, granulin	-1.94	-2.14
HPN, hepsin serine protease	-2.02	-2.27
LPHN1, latrophilin 1	-1.90	-2.38
PRND, prion protein 2	-2.01	-2.50
HYPM, huntingtin interacting protein M	-2.18	-3.98

**Figure 2:**
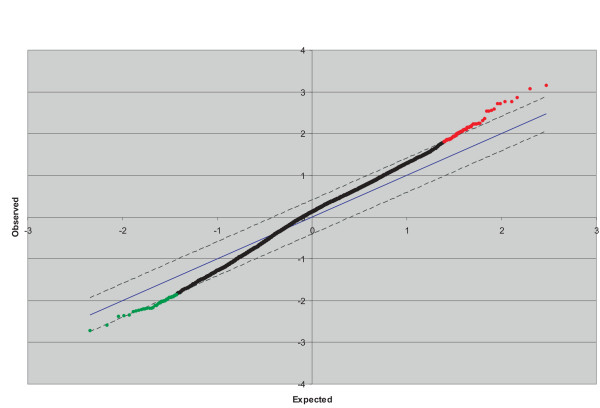
**Two-class analysis of the microarray data set**. The six 293 cells arrays (three from the control clones, and three from the SMC3-overexpressing clones) were subjected to SAM two-class unpaired analysis where the software created a field of observed versus expected gene regulation values from the array data. A delta parameter (0.409) was chosen to limit the field and provide the optimal output of significantly to falsely significantly regulated genes (16 in our analysis). The threshold was set at 1.00 corresponding to a twofold difference in regulation from the control cells when data are entered as log_2 _values. Dashed lines: delta parameter which defines the significance field; dots above the upper line: probable significantly up-regulated genes; dots below the lower line: probable significantly down-regulated genes.

To ensure the accurate identification of genes whose regulation is altered in response to SMC3 overexpression, the array data were normalized and filtered to exclude gene displaying high inter-array variability [18] and then statistically analyzed with SAM [19] (fig. 2). The algorithm uncovered 114 genes that either increase (n = 70) or decreased (n = 44) their expression level by at least two-folds. Differentially expressed genes with an entry in the Unigene database were classified according to their putative main function and listed in Table I. About one-fifth of the affected genes encode secreted or cell surface proteins such as components of the extracellular matrix or cell surface receptors and growth factors. This group of genes includes G*lypican 3 *a heparan sulfate proteoglycan that is a cell surface co-receptor for heparin-binding growth factors and is elevated in several forms of cancer [20]. About two-third of the differentially expressed genes is engaged in signal transduction and gene transactivation. In this group there are genes involved in cAMP signal transduction such the adenylate cyclase 2, CREM and CRE-BPa [21,22]. Further examination of the list of the affected genes reveals a rather specific effect of SMC3 on the expression of genes that are effectors (RhoB and RAB40B) or modulators (ARHGEF4, RGS14, RIN3, PDE6B, ADP-rybosyltransferase) of the ras-rho/GTPase signaling pathway [23-25]. Rho is a family of small GTPases that is not mutated in cancer and therefore their involvement in tumorigenesis is dependent upon the activation status and the expression level. The up-regulation of RhoB expression may be of particular relevance in the context of SMC3-mediated tumorigenesis. RhoB is an early response gene that is transcriptionally activated following DNA damage [26] through a mechanism requiring ATF2-mediated gene transactivation [27]. ATF2 is a member of the CRE-binding protein gene family whose transcriptional activity is in turn directly regulated by ATM in response to genotoxic stress [28]. Interestingly an ATM-dependent pathway is also responsible for the phosphorylation of SMC3 in response to irradiation [3] raising the possibility that RhoB and SMC3 are members of the same DNA integrity surveillance pathway. Other findings suggest that SMC3 and RhoB expression may be linked. The expression of these genes is increased following activation of the Wnt/β-catenin/TCF4 transactivation [6,29]. On the other hand SMC3 and RhoB expression is negatively affected by Ras [30,31]. RhoB has both a positive and negative role in cell growth and it has been postulated it operates in a contextual manner. For example RhoB is essentially required for Ras-mediated transformation but at the same time it is necessary for the apoptotic response of transformed cells to DNA-damaging agents [25-27], suppresses tumorigenesis and restrains the transformed characteristics of neoplastic cells [32]. SMC3-transformed cells however do not display sign of apoptosis as revealed by Annexin V binding or propidinium iodine nuclear staining (data not shown). Furthermore, as revealed by the microarray analysis, the expression of apoptotic agents and mediators was not affected by SMC3 upregulation (Table I). Given that RhoB and SMC3 may exert opposite effect on cell transformation, we postulate that SMC3 oncogenic activity prevails by overcoming the pro-apoptotic action of RhoB.

### SMC3 acts as an oncogene in human cells and activates the expression of a set of early-response genes

**Figure 3:**
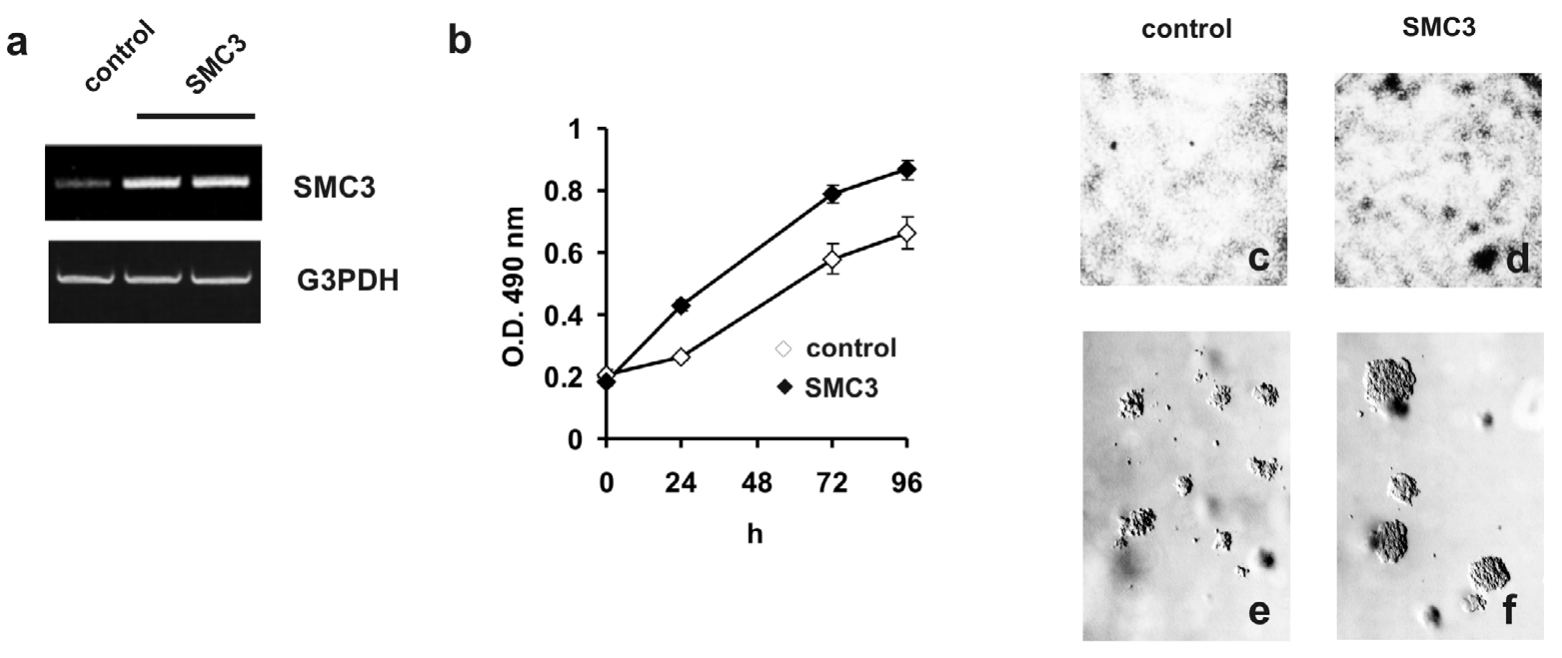
**Effect of SMC3 overexpression on 293 cells colony growth**. a): 293 cells at 70% confluence were transfected with 1 μg/ml of pcDNA3.1 (control) or SMC3-pcDNA3.1 expression vectors and cell RNA collected after 48 hrs in 1 ml TriReagent. SMC3, and G3PDH transcript levels were assayed by semiquantitative RT-PCR and the products analyzed by agarose electrophoresis. The result of a control and two independent samples of cells transfected with SMC3-pcDNA3.1 are shown. b) Growth rate of cells overexpressing SMC3. Cells were seeded in 96-well plates (2,500 cells/well in a final volume of 150 μl) in DMEM medium supplemented with 1.5% FCS. Cell proliferation was assessed by using a CellTiter 96 colorimetric assay. Each point represent the mean ± SD of four independent determinations. c,d) Transfected cells were seeded in 35 mm plates and grown for 10 days in DMEM supplemented with 1.5% FCS. To detect foci of transformation, cells were fixed in 70% ethanol followed by staining with 0.1% methylene-blue. e,f) Colony formation in semisoft agarose. Twenty-four h after transfection, cells were trypsinized and resuspended in 0.2% agarose in DMEM containing 10% fetal bovine serum and plated on top of solidified agarose (0.4%) dissolved in the same medium in 35 mm dishes. After 3 weeks of culture cell colonies were examined under a light microscope.

In order to assesses whether changes in the gene expression observed in cell stably overexpressing SMC3 are part of an early response to SMC3 elevation and may thus mediate the tumorigenic potential of this cohesin protein, 293 cells were transiently transfected with SMC3-pcDNA3.1 or alternatively with the empty expression vector (fig. 3a). Ectopic expression of SMC3 in 293 cells enhanced cell proliferation (fig. 3b) and promoted cell aggregation, rounding and piling as observed when the cells were cultured in serum-deficient medium (fig. 3c,d). As anchorage-independent growth is considered to be *in vitro *test for tumorigenesis, we examined the growth of SMC3-transfected 293 cells in a semi-soft agarose medium. Scoring of the number and the size of the colonies formed revealed that SMC3 acts as an inducer of cell transformation. Not only the percent of colonies formed was increased (~95% compared to ~50% originating from pcDNA3.1-transfected cells) but the colonies formed after three weeks where significantly larger (12 ± 3 colonies of diameter of >100 μm/9 mm^2 ^vs. none generated by pcDNA3.1-transfected cells) (fig. 3e,f). These results demonstrate that SMC3 has the ability to enhance the tumorigenic potential of immortalized human cells. SMC3 does not require the ectopic co-expression of other oncogenes to achieve its tumorigenic function.

**Figure 4:**
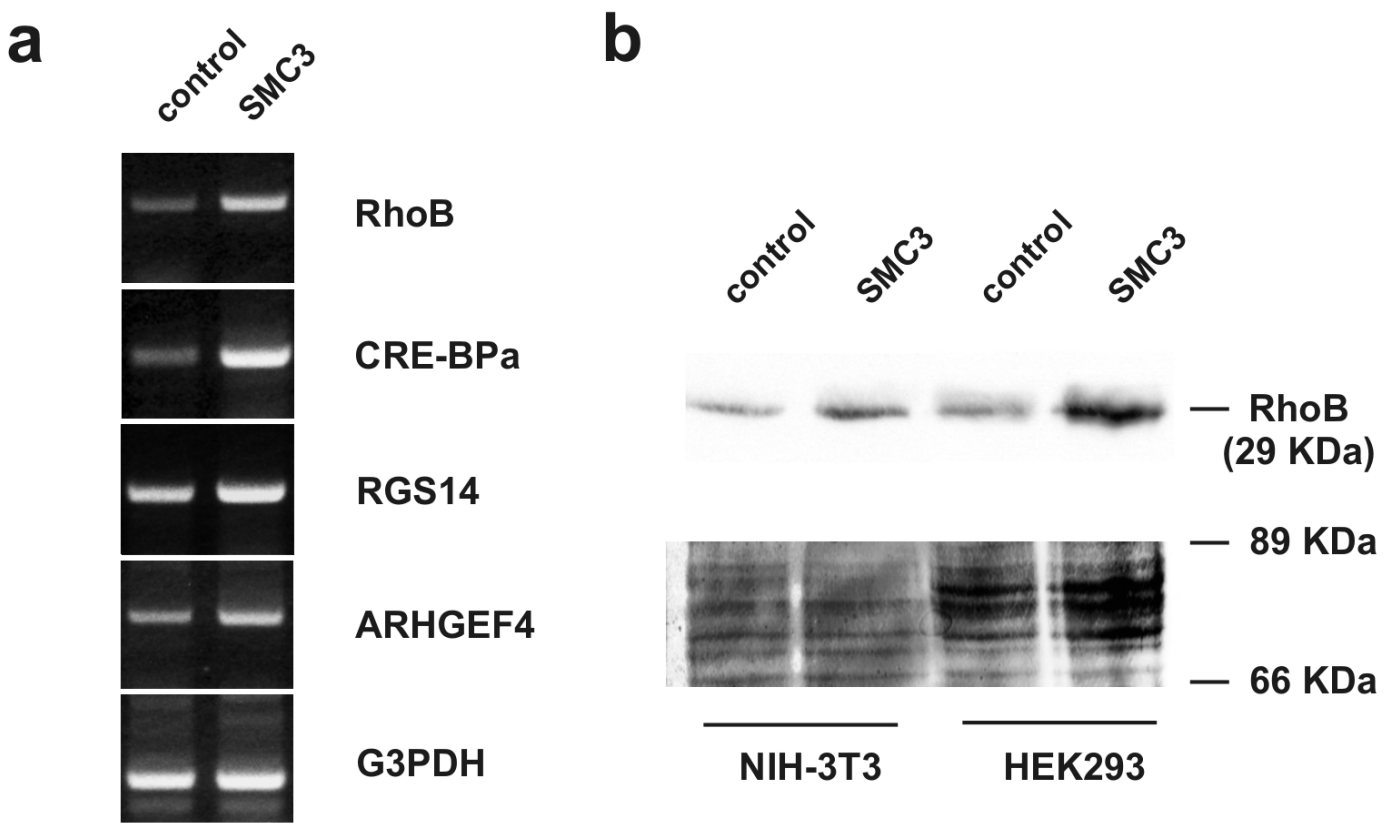
**Effect of SMC3 on the expression of a set of early-response genes**. a) Cells were transfected with 1 μg/ml of pcDNA3.1 (control) or SMC3-pcDNA3.1 and RNA collected after 48 hrs in 1 ml TriReagent. Gene transcripts were amplified by RT-PCR and the products analyzed by agarose electrophoresis. b) Analysis of RhoB level in NIH-3T3 and 293 cells overexpressing SMC3. Cells were transfected as in a) and 48 h later solubilized in lysis buffer. Twenty and 50 μg respectively of NIH-3T3 and 293 cell lysate proteins were analyzed by 12.5% SDS-PAGE. After transfer to nitrocellulose, RhoB was immunodetected using a rabbit polyclonal antibody followed by ECL detection of the immunocomplex. Nitrocellulose-bound proteins were stained with Ponceau-red to evidence the amount of proteins loaded.

**Figure 5:**
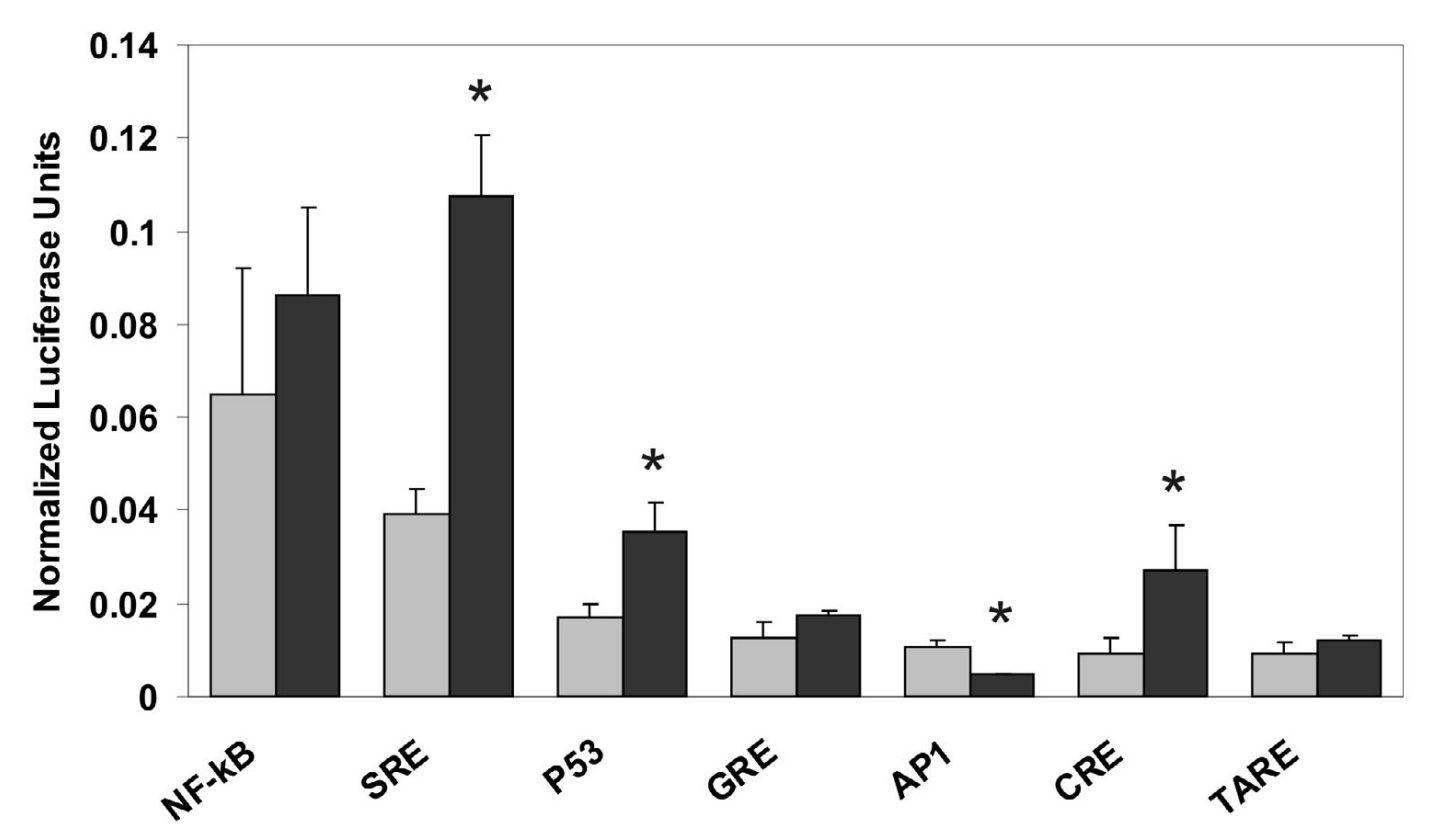
**Transcriptional activity in control and SMC3 overexpressing cells**. Cells were seeded in 12 well plates and used at 70% confluence. Treatment groups, each as triplicate samples, were transfected with 0.1 μg/ml of the indicated reporter vector together with 0.01 μg/ml of PH-RL transfection control vector using 5 μl/ml Lipofectamine. The reporter vectors harbor multiple repeats of the consensus sequence for different transcriptional binding sites which drive the expression of a *firefly *luciferase. Transactivation activity was calculated based on the *firefly *luciferase level correcting for the transfection efficiency using the *renilla *luciferase level in a dual luciferase assay. Data shown are the mean ± SD from three independent determinations. * p < 0.01 SMC3-overexpressing vs. control.

Cells transiently transfected with SMC3-pcDNA3.1 displayed at 3-folds increase in SMC3 transcript level. This was matched by a 5-fold elevation of RhoB transcript level (fig 4a). The effect of SMC3 was confirmed when RhoB protein level was assessed by immunoblotting (fig. 4b). Because SMC3 has the capability to transform NIH3T3 cells [5] we examined whether the ectopic expression of SMC3 enhances RhoB expression in the murine fibroblasts. The Western immunoblotting results confirmed this hypothesis (fig. 4b). The finding corroborates the idea that a causal link exist between SMC3 and RhoB expression that is not restricted to a specific cell context. The mechanism whereby SMC3 elevation leads to RhoB upregulation can be only presently speculated. It has been reported that interference with the cohesin complex turnover, such as it may occur when SMC3 level is elevated, causes derangement of the cell cycle progression and increases the number of chromosomal segregation errors leading to DNA breakage and aneuploidy [33]. As discussed previously, the ensuing activation of the ATM pathway may lead to *RhoB *transactivation via a CREB-dependent mechanism [27,28]. The evidence that p53- and CRE-mediated gene transactivation are enhanced in cells constitutively overexpressing SMC3 (fig. 5), is consistent with an activation of the ATM-dependent pathway [34] and corroborates this scenario. The upregulation of MLH3 and DNMT2 (see Table I) – two genes involved in DNA base mismatch repair, further supports this hypothesis. DNA damage would normally result in cell cycle arrest. Cells constitutively overexpressing SMC3 however display enhanced growth rate suggesting that they can escape the surveillance of the cell cycle gatekeepers. Recently SMC3 has been identified as a component of a complex including SMC1 and BRCA1, that operates as an effector in the ATM-dependent S-phase checkpoint [3,35]. It is possible that when present in excess SMC3 acts in dominant-negative fashion with respect to the activity of the SMC1/SMC3/BRCA1 complex affecting the efficacy of the ATM-dependent S-phase checkpoint.

Further analysis of SMC3-transfected 293 cells and the parent cells provided evidence that in addition to RhoB also CRE-BPa, RGS14 and ARHGEF4 are part of the set of genes activated following transient elevation of SMC3. RGS14 is a target of the p53 tumor suppressor and its overexpression inhibits both Gi- and Gq-coupled growth factor receptor mediated activation of the mitogen-activated protein kinase signaling pathway in mammalian cells [36]. Because p53 transactivation pathway is activated following SMC3 overexpression (see below) we postulate that RGS14 may be involved in counteracting the SMC3 mitogenic activity. ARHGEF4 (also known as Asef) is a Rac-specific guanine nucleotide exchange factor that is activated following binding to APC [37,38]. APC-ARHGEF4 complex plays an important role in the regulation of the actin cytoskeleton, cell morphology and migration and affects E-cadherin-mediated cell-cell adhesion. Agents such as ARHGEF4 may thus act as mediators of the effect of SMC3 overexpression on cell growth and morphogenesis.

### SMC3 overexpression specifically activates the SRE and CRE transactivation pathways

To further examine the cellular response to SMC3 overexpression, the activation level of a number of transactivation pathways was investigated. We reason that selective changes in transcriptional activity could further sort out the key players mediating the SMC3 biological activity. For this purpose 293 cells were transfected with SMC3 expression vector or pcDNA3.1 vector alone (control group) together with a series of luciferase reporters whose expression is driven by target-specific *cis*-acting elements present in multiple copies in the vector promoter. The serum responsive element (SRE) is a known target of RhoB as well as of other ras-rho/GTPase [25]. Consistent with the idea that elevation of RhoB is functionally significant a 2.8-fold elevation of SRE-transactivation activity was detected in SMC3-overexpressing cells (fig. 5). On the contrary AP1-dependent gene transactivation which is mediated by c-jun, c-fos and ATF2 homo or heterodimers, was significantly suppressed. Given that RhoB, but not other ras-rho/GTPase, suppresses AP1-mediated gene transactivation [30] this result support the idea that following SMC3 elevation, RhoB plays a central role in mediating gene transactivation. Consistent with a major role of the CREB in the SMC3 mediated cell events, the response of the CRE-dependent reporter was greatly increased (2.6-fold) in cells stably overexpressing SMC3. This cAMP responsive element is a bona-fide target for the CRE-BPa transcriptional factor whose expression is significantly increased following SMC3 elevation (Table I, fig. 4a). CRE-BPa is a member of the ATF2 cAMP-binding proteins family that is activated by a variety of kinases including protein kinase A, JNK/SAPK, p38-MAPK, AKT, and calcium-calmodulin-dependent kinases and is involved in tumorigenesis of endocrine tissues and different forms of leukemia [21]. To the activation of the CREB transactivation pathway may also contribute the downregulation of CREM, a gene encoding several spliced products some of which are CRE-transactivation repressors [22]. The glucocorticoid, TGFβ-activin and of NF-kB transactivation pathways are potential target of RhoB or CRE-BPa, and their activity was also tested [26,39,40]. NFkB-mediated transactivation which has been reported to be downregulated by RhoB in NIH3T3 cells [26] was not significantly affected in our experiments suggesting that this regulatory mechanism is either cell context-specific or that SMC3 elicits other changes that counterbalance the RhoB-dependent NF-kB loss of activity. Likewise gene transactivation from the GRE and TARE cis-acting elements was not affected. Taken together the results are consistent with the idea that following SMC3 elevation, SRE- and CRE- mediated gene transactivation is specifically engaged.

## Conclusion

The results presented provide a molecular signature of the changes that occurs in epithelial cells following SMC3 overexpression. In particular we document a selective effect of the ectopic expression of SMC3 on a set of early-response genes such as RhoB and CRE-BPa and on the related transcriptional pathways SRE and CRE that play key roles in tumorigenesis. SMC3 acts as an oncogene in human cells and we show that RhoB and CRE-BPa are part of a set of genes activated following transient SMC3 transfection. These findings provide important initial information on the chain of events occurring following SMC3 overexpression that will allow in future studies to focus on the underlying mechanism of the association between SMC3 deregulation and specific oncogenic pathways.

## Methods

### Establishment of cell lines stably overexpressing SMC3

293 cells were grown to 70% confluence in 10 cm plates and transfected with 3 μg of pcDNA3.1 expression vector harboring the entire human SMC3 coding sequence (SMC3-pcDNA3.1) [5] and using Lipofectamine as transfecting agent. To generate a control cell line a second batch of cells was transfected instead with the empty pcDNA3.1 vector. After 48 h stably transfected cells were selected in medium containing 500 μg/ml of G418. Clones of the surviving cells were expanded and SMC3 expression examined by semi-quantitative RT-PCR. For this purpose, 1 μg of cell RNA was reverse transcribed with Sensiscript (Qiagen) reverse transcriptase priming with oligo-dT. An aliquot of the RT reaction product was amplified using ExTaq (Takara) DNA polymerase and SMC3-specific primers of sequence: 5'-GAGTAGAAGAACTGGACAGA-3' and 5'-GATTGTACCTCAGTTTGCTG-3'. To ensure that the amplification reaction had not reached saturation, DNA production was monitored after 25 and 30 cycles by analysis on 1% agarose and by staining with ethidium bromide. Gels were photographed, the picture scanned and the band intensity quantified by densitometry with an image scanner. SMC3 protein expression was assessed by Western immunoblotting. Briefly, cell lysates in 150 mM NaCl 1% Nonidet P40 0.5% Na-deoxycholate 50 mM Tris-HCl pH 7.4 were electrophoresed on 8% SDS-PAGE slab gel and the separated proteins transferred onto a nitrocellulose filter. Immunoblotting was performed with goat anti-human SMC3 (1:1,000) antibody (Santa Cruz Biotech) at 25°C for 1 h, followed by incubation in anti-goat IgG horseradish peroxidase conjugated (1:10,000) secondary antibody. Immunocomplexes were identified using an enhanced chemiluminescence (ECL) kit (Pierce) followed by autoradiograph. Three SMC3 overexpressing clones and three control clones were selected for the gene expression profiling.

### Microarrays and data analysis

For the target preparation, 5 μg of human untransfected and transfected 293 cell line total RNA were reverse transcribed with SuperscriptT-II/RNaseH^- ^priming with T7-(dT)24 oligonucleotides and the second-strand cDNA synthesized using E. coli DNA polymerase I [18]. Biotinylated cRNA was generated using T7 RNA polymerase and Biotin 11-UTP. Ten μg of purified unfragmented target cRNA was used for hybridization of each KCC/TJU human 18.5 K Expression Bioarray (Compugen Human Oligo Set 1.0) chip containing 18,861 oligos (65-mer) corresponding to 17,260 unique clusters and 18 bacterial control probes. The microarrays were hybridized, washed, and processed using a direct detection method of the biotin-containing transcripts by a Streptavidin-Alexa647 conjugate. Processed slides were scanned using a Perkin Elmer ScanArray XL5K scanner and the spot intensity quantitated using the ScanArray Scanning and QuantArray programs (PerkinElmer) [18]. The chips analyzed presented consistent staining over the entire microarray and had appropriate data distribution. Spots with raw intensity value comprised within one SD from the average background value were excluded from the analysis. The remaining values – about 90% of the whole arrayed genes, were normalized by dividing each spot's intensity (after background subtraction) by the median signal intensity of all test probes. Genes that displayed inter-array variability exceeding one SD unit were furthermore excluded from the final statistical analysis. The data and protocols have been submitted to the EBI ArrayExpress database (samples 171479SUB800 through 171484SUB800).

### Statistical analysis of the microarray data

We input the log_2 _of the gene expression measurements from three sets of microarray experiments each including a control and an SMC3 overexpressing cell sample. Through a series of permutation the program computes a statistic score *di *for each gene *i *measuring the strength of the relationship between gene expression and the response variable and creates a profile of observed versus expected values. The values which lie outside a user-defined region that can be adjusted to achieve an optimum of positive vs. false positive values, are considered significantly related to the response and thus regarded as significantly regulated genes (see fig. 2). Since each experiment consisted of the data from two independent chips hybridized with the control and the SMC3-overexpressing cells cDNA, an unpaired two-class analysis was carried out to discover significant changes in gene regulation compared to the control cell line.

### Gene transactivation activity assay

Reporter vectors harboring multiple copies of the consensus sequences for the AP1, cAMP (CRE), serum (SRE), p53, TGFβ (TARE), NF-kB and glucocorticoid (GRE) response elements, were obtained from Stratagene or Clontech. Cells cultures at 70% confluence in 12 wells plates were used in all the experiments. The transfection mix contained 10 ng/ml of phRL-SV40 plasmid to monitor the transfection efficiency, 100 ng/ml of the designed plasmid, and 1 μg/ml of SMC3-pcDNA3.1 expression vector. Plasmids were mixed in medium 199 followed by the addition of 10 mg/mg DNA of Tfx-50 (Promega) transfection agent according to the manufacturer directions and finally added to the cultures. After 1 h incubation at 37°C in humidified incubator the cells were supplemented with 2 ml of growth medium and the luciferase activity assayed 24 h later using a Promega dual-luciferase kit. All experiments were carried out with triplicate samples. The statistical difference between groups of data was analyzed by Student's *t*-test.

### SMC3 transient transfection and gene transcript level analysis

Cells (either 293 or NIH3T3) at 70% confluence were transiently transfected with 1 μg/ml of SMC3-pcDNA3.1 expression vector using Lipofectamine as transfection agent. Control cells were transfected instead with 1 μg/ml of pcDNA3.1 empty vector. After 48 h, the cells were washed in PBS and the total RNA extracted with TriReagent. Gene transcripts were amplified by RT-PCR and the products quantified by gel electrophoresis. The primers used had the following sequence: RhoB: 5'-CCTGCTGATCGTGTTCAGTAA-3' and 5'-TCATAGCACCTTGCAGCAGTT-3'; CRE-BPa: 5'-ATGATTTATGAGGAATCCAAGAT G-3' and 5'-TTAAAGAATCGGATTCAGGTCTGT-3'; RGS14: 5'-CTGGTGGGCAATGAACAGAAGGCC-3' and 5'-GGGCTGAGTCGGTGGTGGAGTTCA-3'; ARHGEF4: 5'-AGCCTCAAGCCAAAAGCCAGCAGC-3' and 5'-CTCACTTGCTGGCAGAGGAAGGCCA-3'; G3PDH: 5'-TGAAGGTCGGAGTCAACGGATTTGGT-3' and 5'-CATGTGGGCCATGAGGTCCACCAC-3'. RhoB protein level in cells was examined by Western immunoblotting as described previously using a rabbit polyclonal antibody (1:1,000) from Bethyl.

### Cell proliferation assay

Cell proliferation was examined using a CellTiter 96 (Promega) assay kit according to the manufacturer's instructions. Cells growing in log phase were trypsinized and seeded in 96-well plates (2,500 cells/well in a final volume of 150 μl) in replicates of 4 and incubated at 37°C in 5% CO_2 _and 95% air in DMEM medium containing 1.5% FCS. At 24 h, 72 h or 96 h, 20 μl of the kit dye solution was added to each well and the plates incubated at 37°C for an additional 1 h. The absorbance of the formazan product generated was measured at 490 nm using a 96-well Dynatech MR600 plate reader.

### Cell overgrown assay

In order to examine the ability of cells to form foci of transformation, cells were seeded at 30% confluence in 35 mm plates and cultured in DMEM supplemented with 1.5% medium. After 7 days the medium was removed, the cell washed with PBS, and fixed with 70% ethanol on ice. Foci of cell aggregation were evidenced by staining with 0.1% methylene-blue dissolved in water followed by 3 washing in water to decrease background staining.

### Anchorage-independent cell growth in soft agar

Anchorage-independent colony formation of cells was assayed as described [5]. Briefly, cells growing in log-phase were trypsinized and resuspended at 37°C in 0.2% agarose in DMEM containing 10% fetal bovine serum and plated on top of solidified agarose (0.4%) dissolved in the same medium in 35 mm dishes. After 3 weeks of culture at 37°C in CO_2 _humidified incubator, the number of cell aggregates over that of single cells and the number of colonies of diameter >100 μm found in randomly selected areas of 9 mm^2^, was recorded.

## Authors' contributions

The experiments with cells were conducted in G. G. laboratory with the technical help of Mr. Amit Agrawal and Mr. Chirag Patel. G.G. is also responsible for the drafting of the manuscript. The microarray analysis was conducted by C-G.L at the Microarray Facility of the Kimmel Cancer Center.
